# Hepatoprotective Effects of *Lactobacillus* on Carbon Tetrachloride-Induced Acute Liver Injury in Mice

**DOI:** 10.3390/ijms19082212

**Published:** 2018-07-29

**Authors:** Xiaoyong Chen, Jing Zhang, Ruokun Yi, Jianfei Mu, Xin Zhao, Zhennai Yang

**Affiliations:** 1 Beijing Advanced Innovation Center for Food Nutrition and Human Health, Beijing Technology & Business University (BTBU), Beijing 102488, China; chenxiaoyong522@163.com; 2 Chongqing Collaborative Innovation Center for Functional Food, Chongqing University of Education, Chongqing 400067, China; yirk@cque.edu.cn (R.Y.); mujianfei@foods.ac.cn (J.M.); 3 Department of Food Science and Technology, South China University of Technology, Guangzhou 510640, China; 4 Department of Environmental and Quality Inspection, Chongqing Chemical Industry Vocational College, Chongqing 401220, China; zhangjing@foods.ac.cn

**Keywords:** *Lactobacillus fermentum*, *Lactobacillus plantarum*, hepatoprotective effect, CCl_4_

## Abstract

The aim of this study was to investigate and compare the effects of heat-killed and live *Lactobacillus* on carbon tetrachloride (CCl_4_)-induced acute liver injury mice. The indexes evaluated included liver pathological changes, the levels of alanine aminotransferase (ALT), aspartate aminotransferase (AST), superoxide dismutase (SOD), glutathione (GSH), and malondialdehyde (MDA) in the serum, related gene expression (*IL-1β*, *TNF-α*, *Bcl-2,* and *Bax*), and related proteins levels (Bax, Bcl-2, Caspase 3, and NF-κB p65). Compared with the model group, the results indicated that the levels of ALT, AST, and MDA in the serum, the expression levels of *IL-1β*, *TNF-α,* and *Bax*, and the protein levels of Bax, Caspase 3, and NF-κB p65 significantly decreased, and the pathologic damage degree all significantly reduced after live *Lactobacillus fermentum* (L-LF) and live *Lactobacillus plantarum* (L-LP) treatment. Additionally, the levels of SOD and GSH in the serum, the gene expression of *Bcl-2*, and the protein level of Bcl-2 significantly increased after L-LF and L-LP treatment. Although HK-LF and HK-LP could also have obvious regulating effects on some of the evaluated indexes (ALT, AST, the expression levels of *TNF-α* and *Bax*, and the protein level of Bcl-2) and play an important role in weakening liver damage, the regulating effects of L-LF or L-LP on these indexes were all better compared with the corresponding heat-killed *Lactobacillus fermentum* (HK-LF) and heat-killed *Lactobacillus plantarum* (HK-LP). Therefore, these results suggested that LF and LP have an important role in liver disease.

## 1. Introduction

The liver plays an important role in maintaining organism health, and it is involved in metabolism, detoxification, hematopoiesis, immunity, biliation, and liver regeneration. Meanwhile, it is also vulnerable to a series of stimuli, including viruses, toxins, drugs, alcohol, and trauma, which could all ultimately lead to acute or chronic liver damage [1,2]. Clinical data have shown that long-term liver damage can result in liver fibrosis, cirrhosis, and hepatocellular carcinoma. Thus, it is of important significance to find a way to prevent liver damage [3]. At present, probiotics are being widely studied as a new therapy idea. Increasing evidence has demonstrated that probiotics exert beneficial effects on the health of the host. For example, *Lactobacillus plantarum* can maintain the growth rate of infant mice during chronic malnutrition [4]. *Lactobacillus* GG exerts antidiabetic effects on diabetic rats [5]. Additionally, *Lactobacillus casei* can maintain host intestinal homeostasis, and exerts anti-inflammatory effects [6]. However, most of the research of *Lactobacillus* has focused on the physiological function of a live strain and its metabolites. These were lacking in a comparison of the physiological functions between live and dead strains. Therefore, probiotic research has often been questioned as to whether the entire effect is due to cellular components, or whether there are further advantages provided by live probiotic bacteria [7].

At present, many different animal models of liver injury have been developed. For example, thioacetamide (TAA) can lead to liver injury by interfering RNA transport from the nucleus into the cytoplasm and protein synthesis, and lead to hepatocyte necrosis by damaging the hepatocyte membrane [8]. Concanavalin A (Con A) can promote mitosis and induce T-cell mediated liver damage, and its feature is similar to the liver damage of autoimmune hepatitis [9]. Besides, the model is also induced by CCl_4_ and d-galactosamine. In this study, the liver injury model was induced by CCl_4_. CCl_4_ is a compound with hepatotoxicity to liver cells, and researchers have used it to induce various hepatic injuries in animal models to elucidate the protection mechanisms to liver injury or hepatic toxicity mechanisms [10]. At present, it is generally accepted that the mechanism of CCl_4_-induced hepatic lesion formation is based on the formation of free radicals and a resulting peroxide chain reaction. CCl_4_ is activated by hepatic cytochrome P450 after entering the body, and then forms unstable trichloromethyl and trichloromethyl peroxyl radicals. These two kinds of free radicals can eventually lead to hepatic lesions by attacking polyunsaturated fatty acids in biofilm phospholipids and trigger lipid peroxidation reactions [11]. Based on CCl_4_-induced hepatic injury animal models, the study of the hepatoprotective effect of microorganisms has made some progress [12,13,14].

Moreover, the existing research has shown that *Lactobacillus* has a good ability to provide antioxidants. For example, *Lactobacillus casei* [15], *Lactobacillus fermentum* [16], *Lactobacillus plantarum* [17], *Lactobacillus rhamnosus* [18], and *Lactobacillus acidophilus* all create antioxidants [19]. Therefore, we assumed that *Lactobacillus* would confer a protective effect on CCl_4_-induced acute liver injury in this study. Moreover, heat-killed probiotics provide advantages for industrial production and consumer usage, including high safety, convenient storage and transport, and shelf-life extension relative to live strains [20]. Therefore, we wanted to know the difference between the effects of heat-killed and live *Lactobacillus* on liver damage.

In this study, *Lactobacillus fermentum* (LF) and *Lactobacillus plantarum* (LP) were isolated from Chinese pickles from a local farmer’s home (Nan’an District, Chongqing, China) using traditional pure culture techniques. The two strains were deposited in the China General Microbiological Culture Collection Center. Previous works proved that LF and LP had excellent resistance to low pH and bile salt. The aim of the present study was to investigate and compare the hepatoprotective effects of heat-killed and live *Lactobacillus* using a CCl_4_-induced acute liver injury murine model.

## 2. Results

### 2.1. Morphological Characteristics

**Figure 1:**
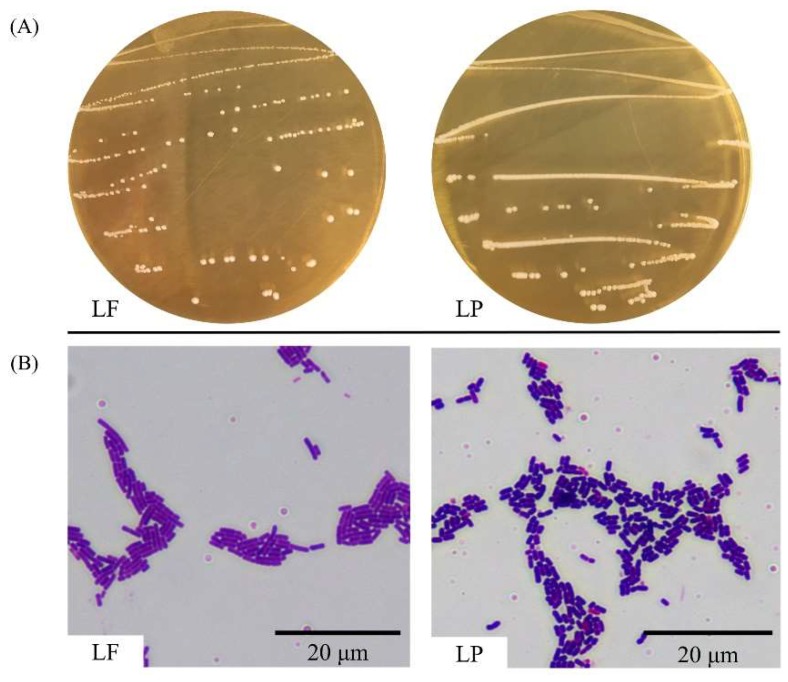
Morphology of strains. (**A**) Colony morphology; (**B**) Cell morphology.

As shown in Figure 1A, the colonies of LF and LP were all white, opaque, round, smooth on the surface, convex in the center, and had neat edges. As shown in Figure 1B, the cells of LF and LP were all rod-shaped, but they were not all identical in size. Gram staining showed that LF and LP were both gram-positive bacteria.

### 2.2. Species Analysis

**Figure 2:**
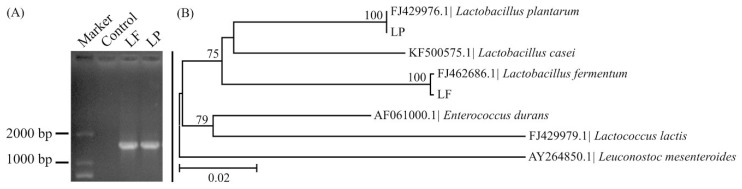
16S rDNA sequence analysis. (**A**) Detection of PCR product using agarose gel electrophoresis; (**B**) Species analysis using a phylogenetic tree of 16S rDNA sequence.

After detection using agarose gel electrophoresis, 16S rDNA PCR products from LF and LP were satisfactorily visible with a clear band at the correct place (approximately 1500 bp) and without non-specific amplification (Figure 2A). Based on a phylogenetic tree of 16S rDNA sequences, LF grouped together with *Lactobacillus fermentum* in a branch, and LP grouped together with *Lactobacillus plantarum* in a branch, the homologies were all 100%. Therefore, LF was considered to be *Lactobacillus fermentum*, and LP was considered to be *Lactobacillus plantarum*. In addition, the LP branch grouped together with the LP branch in a new branch, and the homology was 75% (Figure 2B).

### 2.3. Tolerance to Stimulated Gastric Juice and Bile Salt

**Figure 3:**
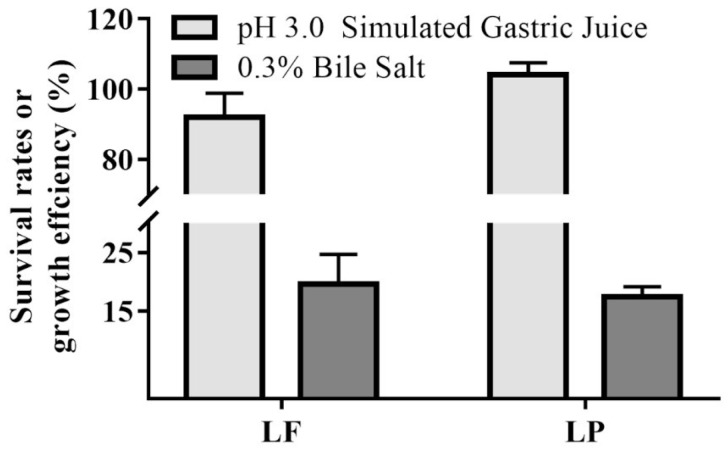
Effects of stimulated gastric juice and bile salt on strains.

The tolerance to gastric juice and bile salt were a precondition of developing and keeping the physiological functions of a probiotic in the body. Therefore, we evaluated the survival rates of LF and LP after stimulated gastric juice treatment and the growth efficiency of LF and LP after bile salt treatment in vitro. As shown in Figure 3, LF and LP both exhibited good tolerance to simulated gastric juice, and their growth efficiencies after 0.3% bile salt were also high.

### 2.4. Pathological Observation

**Figure 4:**
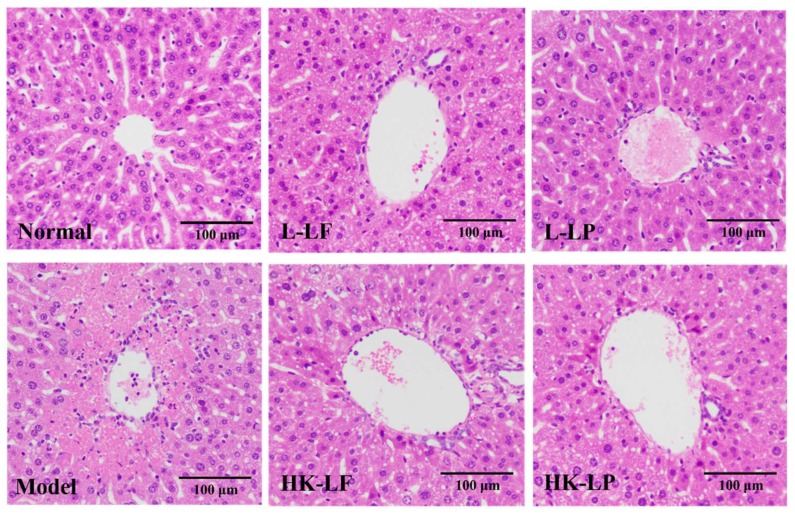
Histopathology observation of liver.

The pathologic changes in stained sections were observed under a microscope to evaluate the effects of LF and LP treatment. As is evident from Figure 4, liver cells were intact, their structures were clear, and they were uniformly distributed in the normal sections, but in the model sections, the section showed severe hepatocellular necrosis and inflammatory cell infiltration, and the liver cells had no regular arrangement around the central veins. After L-LF or heat-killed *Lactobacillus fermentum* (HK-LF) and L-LP or heat-killed *Lactobacillus plantarum* (HK-LP) treatment, their pathologic damage degree was reduced compared with those of the model samples. In addition, the damage degrees of L-LF and L-LP were obviously lower than those of HK-LF and HK-LP.

### 2.5. Measurement of ALT and AST

**Figure 5:**
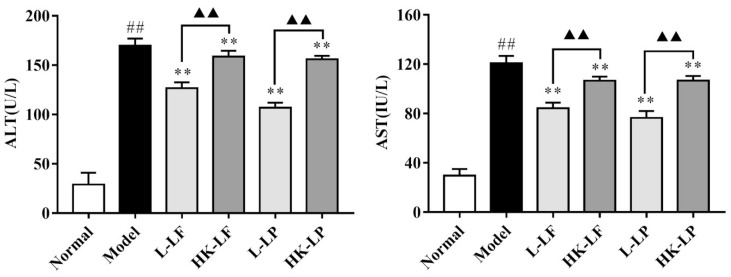
The levels of liver enzymes in serum. ^##^ *p* < 0.01 compared with the normal group, ** *p* < 0.01 compared with the model group, and ^▲▲^ *p* < 0.01.

To evaluate the effects of LF and LP on CCl_4_-induced liver dysfunction, the liver enzymes in serum that are related to liver function were analyzed. As shown in Figure 5, the levels of alanine aminotransferase (ALT) and aspartate aminotransferase (AST) in the model group significantly increased compared with those in the normal group. Compared with the model group, the levels of ALT and AST were significantly reduced in the L-LF, L-LP, HK-LF, and HK-LP groups. In addition, compared with HK-LF and HK-LP, the corresponding live group is more significant.

### 2.6. Measurement of SOD, GSH, and MDA

**Figure 6:**
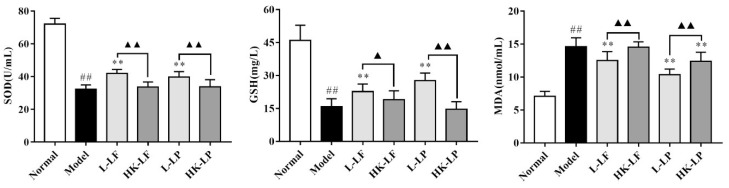
The levels of antioxidant indexes in serum. ^##^ *p* < 0.01 compared with the normal group, ** *p* < 0.01 compared with the model group, ^▲^ *p* < 0.05 and ^▲▲^ *p* < 0.01.

Oxidative stress is closely related to liver damage, so we measured the levels of superoxide dismutase (SOD), glutathione (GSH), and malondialdehyde (MDA) in the serum, because these indicators are key markers of oxidation. As shown in Figure 6, compared with the results of the normal group, the levels of SOD and GSH in the model group were significantly reduced, and the level of MDA in the model group was significantly increased. However, compared with the model, L-LF can significantly increase the level of SOD and significantly reduce the level of MDA. L-LP can significantly increase the levels of SOD and GSH and significantly reduce the level of MDA, and HK-LP can also significantly reduce the level of MDA. Compared with the heat-killed group, the regulated effects of the live group are more significant.

### 2.7. RT-qPCR

**Figure 7:**
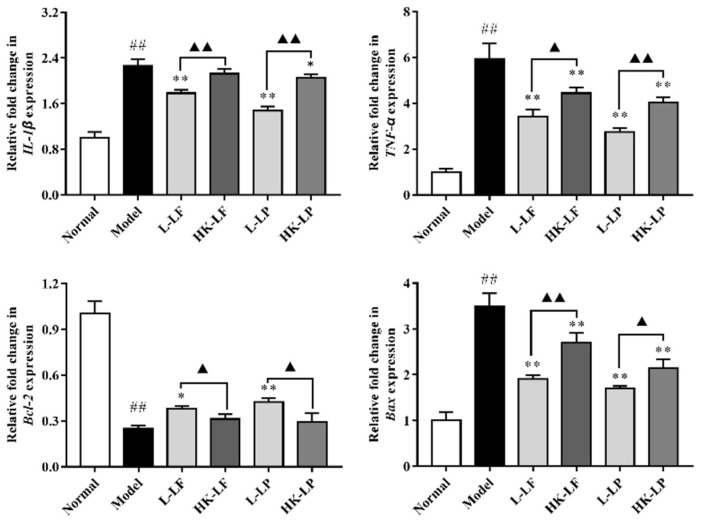
Related gene expression levels in liver injury. ^##^ *p* < 0.01 compared with the normal group, * *p* < 0.05, ** *p* < 0.01 compared with the model group, ^▲^ *p* < 0.05 and ^▲▲^ *p* < 0.01.

As shown in Figure 7, compared with the normal group, the expression levels of *IL-1β*, *TNF-α*, and *Bax* in the model group were significantly increased, and the expression level of *Bcl-2* in the model group was significantly reduced. However, compared with the model group, L-LF and L-LP both significantly reduced the expression levels of *IL-1β*, *TNF-α,* and *Bax*, and L-LP significantly increased the expression level of *Bcl-2*. Moreover, HK-LF and HK-LP also both significantly reduced the expression levels of *TNF-α* and *Bax*, and HK-LP significantly reduced the expression level of *IL-1β*. Most notably, compared with the heat-killed treatment groups (HK-LF and HK-LP), the corresponding live groups (L-LF and L-LP) significantly reduced the expression levels of *IL-1β*, *TNF-α,* and *Bax*, and could also significantly increase the expression level of *Bcl-2*.

### 2.8. Western Blot

**Figure 8:**
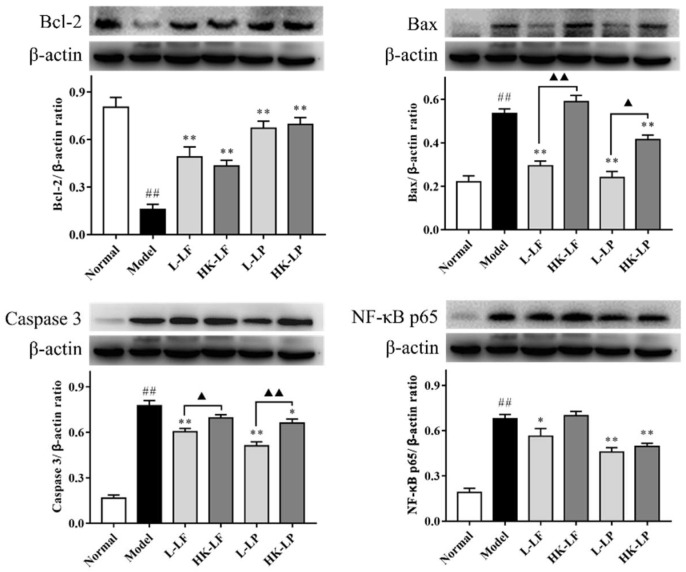
Related protein levels in liver injury. ^##^ *p* < 0.01 compared with the normal group, * *p* < 0.05, ** *p* < 0.01 compared with the model group, ^▲^ *p* < 0.05 and ^▲▲^ *p* < 0.01.

Bcl-2, Bax, Caspase 3, and NF-κB p65 protein levels as determined by Western blot analysis are shown in Figure 8. Compared with the results of the normal group, the protein level of Bcl-2 in the model group was significantly reduced, and the protein levels of Bax, Caspase 3, and NF-κB p65 in the model group were significantly increased. However, compared with the model group results, the protein level of Bcl-2 significantly increased, and the protein levels of Bax, Caspase 3, and NF-κB p65 significantly reduced after L-LF, L-LP, and HK-LP treatment. In addition, HK-LF could also significantly increase the protein level of Bcl-2. Through comparison, we found that the protein levels of Bax and Caspase 3 in the live groups (L-LF and L-LP) were markedly higher than in the corresponding heat-killed groups (HK-LF and HK-LP).

## 3. Discussion

*Lactobacillus* has many origins, including the feces of infants, the intestinal tract of healthy adults, and fermented foods [21,22]. In this study, LF and LP were isolated from Chinese pickles (Chinese pickles, a popular traditional fermentation product of southwestern China, contain abundant *Lactobacillus*). Generally, resisting gastric juice and bile salts in the gastrointestinal tract is a fundamental requirement of probiotics [23]. Thus, evaluating the ability of LF and LP to simulated gastric juice and bile salt was an essential test. These results could indicate whether experimental strains remained active when they reached the intestine. According to an external virtual model of the gastrointestinal tract, we determined that LF and LP were largely unaffected at pH 3.0 in simulated gastric juice, and could maintain good growth activity in 0.3% bile salts.

Our liver injury model, which was induced by CCl_4_, is the most commonly used animal model for evaluating the hepatoprotective effects of plant extracts, drugs, and probiotics [16,24]. In our study, we found that CCl_4_ treatment resulted in severe hepatocellular necrosis, inflammatory cell infiltration (lymphocytes and macrophages), and liver cells with no regular arrangement around the central veins. However, the pathologic degree of damage was reduced after L-LF or HK-LF and L-LP or HK-LP treatment; thus, they played a positive role in protecting against liver damage.

In addition, we also found that the levels of AST (Aspartate aminotransferase) and ALT(Alanine aminotransferase) in the serum were significantly decreased after L-LF or HK-LF and L-LP or HK-LP treatment. AST and ALT are sensitive markers of liver damage. ALT is mainly distributed in the cytoplasm of hepatocytes, and the increased level of ALT in the serum reflected the damage of liver cell membranes. AST is mainly distributed in the cytoplasm and mitochondria of hepatocytes, and the increased level of ALT showed that the damage of hepatocytes reached the level of organelles [25]. These results were consistent with previous studies [26]. Reduction in two different types of liver enzymes implied that LF and LP treatment could prevent liver damage. HK-LF and HK-LP could also down-regulate the increased levels of AST and ALT after CCL_4_ treatment. Therefore, the dead cells in LF and LP treatments also exerted beneficial effects on liver damage.

The changes in the levels of serum SOD, GSH, and MDA showed that LF and LP could effectively decrease oxidative stress reactions. SOD is one of the potent antioxidant enzymes in cells and can catalyze the conversion of superoxide ions into oxygen and hydrogen peroxide [10]. GSH is a major non-enzymatic scavenger that can regulate intracellular redox homeostasis, and the mechanistic studies on carbon tetrachloride (CCl_4_)-induced liver damage revealed that GSH conjugation played an important role in the clearing of toxic metabolites [27]. Lipid peroxidation is closely related to the pathogenesis of hepatic injury by the free radical derivatives of CCl_4_, and mainly leads to cell membrane damage and the consequent release of marker enzymes of hepatotoxicity [28]. MDA is the final product of lipid peroxidation, so it is a key marker of lipid peroxidation. After L-LF or HK-LF and L-LP or HK-LP treatments, the levels of SOD and GSH significantly increased in L-LF and L-LP treatments, and the level of MDA significantly reduced in L-LF, L-LP, and HK-LP. Therefore, LF and LP treatment had significant antioxidant properties, and the effects were remarkably different between the live group and the heat-killed group.

Apoptosis is an initiated cell death process that is controlled by multiple genes [29]. Generally, apoptosis signaling is caused by multiple stress conditions, including ultraviolet light, oxidative stress, chemical agents, drugs, and viruses. These signals can induce apoptosis, inducing factor generation, activate the Caspase cascade, and ultimately lead to cell apoptosis [30]. Note that apoptosis is controlled by numerous genes. Bcl-2 and Bax are important control factors of apoptosis. Bcl-2 has effects on anti-apoptotic mechanisms, Bax can accelerate cell apoptosis, and Caspase 3 is the central effector of apoptosis. Caspase 3 is used as a reliable indicator to determine the severity of apoptosis. The present study demonstrated that L-LF and L-LP caused the significant up-regulation of *Bcl-2* expression, and L-LF or HK-LF and L-LP or HK-LP could cause the down-regulation of *Bax* expression. We also found that L-LF and L-LP had significant effects on the regulation of *Bcl-2* and *Bax* expression compared with corresponding HK-LF or HK-LP, which were both consistent with previously published work. Additionally, we also found that the protein levels of Bcl-2 significantly increased in L-LF, HK-LF, L-LF, and HK-LP treatments, and the protein levels of Bax and Caspase 3 were significantly reduced in L-LF, L-LF, and HK-LP treatments. The protein levels of Bax and Caspase 3 in L-LF and L-LF treatments were significantly lower than the corresponding heat-killed group.

NF-κB, a nuclear transcription factor, can regulate apoptosis, inflammation, tumorigenesis, and gene expression, and it plays an important role in liver injury [31]. Furthermore, previous research has shown that *IL-1β* and *TNF-α* expression was related to the activation of NF-κB. In this study, we demonstrated that L-LF and L-LP, as well as HK-LP, could all down-regulate the expression levels of *IL-1β* and *TNF-α*, unlike HK-LF. After determining the protein levels of NF-κB p65 by Western blotting, we found that its protein levels were significantly reduced after L-LF, L-LP, or HK-LP treatment. These results showed that L-LF or HK-LF and L-LP or HK-LP had beneficial effects on CCl_4_-induced acute liver injury mouse models to a different degree. As anticipated, the live groups performed better than the heat-killed groups.

The study showed that probiotics had some functional effects in nonalcoholic fatty liver disease, nonalcoholic steatohepatitis, and cirrhosis [32]. HK-LF and HK-LP also showed the liver protective effects in mice. However, this study also has deficiencies. The beneficial effects of HK-LF and HK-LP were not nearly as good as the beneficial effects of the corresponding L-LF and L-LP. However, HK-LF and HK-LP can also have significant effects on some of the related indexes of liver injury (including ALT, AST, and so on), but we did not determine what cellular compounds exerted these beneficial effects. Therefore, further work will expand this research subject.

## 4. Materials and Methods

### 4.1. Strains

Two strains (LF and LP) were used in this study; they were isolated from the Chinese pickle from a local farmer’s home (Nan’an District, Chongqing, China) using traditional pure culture techniques, and deposited in the China General Microbiological Culture Collection Center (CGMCC, Beijing, China). The preservation number of LF is 14493 and the preservation number of LP is 14491.

The purified *Lactobacillus* was incubated at 37 °C for 18 h in de Man, Rogosa, and Sharpe (MRS, Difco Laboratories, Detroit, MI, USA ) broth or 37 °C for 24 h in MRS agar; then, the LF and LP cells were collected by centrifugation (4 °C, 4 000 r/min for 10 min), and cell morphology was observed by gram staining [33].

The genus of the purified strains was analyzed using 16S rDNA sequence and a phylogenetic tree. First, the genome DNA was extracted as a PCR template by TIANamp bacteria DNA kit (Tiangen, Beijing, China), and its 16S rDNA gene was amplified using the following primer sequences (F: 5′-AGAGTTTGATCCTGGCTCA-3′ and R: 5′-CTACGGCTACCTTGTTACGA-3′); then, the amplification product was visualized by using 1.5% agarose gel electrophoresis, and sent for sequencing (TSINGKE, Chengdu, Sichuang, China). Finally, the phylogenetic tree of the 16S rDNA sequence was built using MEGA 5.05 (reference strain sequences were selected from the NCBI database).

### 4.2. Evaluation Gastrointestinal Tolerability in Vitro

A 100-mL aliquot of simulated gastric juice was composed of 0.35 g of pepsin and 0.2 g of NaCl. The solution pH was adjusted to pH 3 with 1 mol/L HCl using a pH meter (pHS-3C, Shanghai Lei Ci Instrument Factory, Shanghai, China) and sterilized by filtering through a 0.45-µm filter (Millipore, Billerica, MA, USA). Then, 10 mL of inocula was centrifuged at 4000 r/min for 10 min, washed twice, and resuspended with an equal volume of sterile saline. Next, 1-mL suspensions were mixed with 9 mL of simulated gastric juice and incubated in a shaking incubator (37 °C, 150 r/min) for 3 h. After that, colony-forming units (CFU) were separately counted at 0 h and 3 h [34]. The survival rate (SR) was calculated using the following equation: SR (%) = (N_3_/N_0_) × 100, where SR is the survival rate of *Lactobacillus*, and N_0_ is the number of viable cells (CFU/mL) at 0 h and N_3_ is the number of viable cells (CFU/mL) at 3 h.

In addition, 100-μL suspensions were inoculated into 5 mL of MRS-THIO (MRS broth plus 0.2% of sodium thioglycolate) with or without 0.3% (*w*/*v*) oxgall. Bacterial cells in the culture broth were measured by reading of the optical density (OD) at 600 nm after 24 h of incubation at 37 °C [35]. The percentage of bile tolerance was calculated using the following equation: BT (%) = (A/A_0_) × 100, where BT is the bile tolerance of *Lactobacillus*, A is the OD of a bacterial cell with 0.3% (*w*/*v*) oxgall, and A_0_ is the OD of a bacterial cell without 0.3% (*w*/*v*) oxgall.

### 4.3. Animal Models and Treatment

Sixty Kunming mice (male, six weeks old) were obtained from the Experimental Animal Center of Chongqing Medical University (Chongqing, China). All of the mice were fed with a standard mice chow diet and water under constant conditions at 25 ± 2 °C in a 12-h light/dark cycle. After adaptive feeding for a week, the 60 mice were divided into six groups according to body weight (each group had 10 mice). The six groups were a normal group (normal), a model group (model), a live LF group (L-LF), a heat-killed LF group (HK-LF), a live LP group (L-LP), and a heat-killed LP group (HK-LP). The experiment lasted 14 days, and an animal model of acute liver injury was induced using 1% CCl_4_ (10 mL/kg·BW, diluted in soybean oil, intraperitoneal injection) on the last day. The heat-killed condition of LF and LP was created by heating in a boiling water bath for 30 min, and the gavage dose of live and heat-killed LF and LP was 10^9^ CFU/kg·BW in each group with *Lactobacillus* treatment. During the experiment, the groups with LF and LP treatment were given the corresponding live and heat-killed *Lactobacillus* solution every day (10^9^ CFU/kg·BW), and the normal and model groups were given the same quantity of normal saline every day. All of the mice were killed at 16 h after injection of CCl_4_, and then, the blood serum was collected (4 °C, 3000 r/min for 15 min), as were liver samples. Finally, a portion of the liver from each mouse was used to perform tissue biopsies, and the surplus liver sample was transferred to an ultralow temperature freezer after liquid nitrogen flash freezing. Blood serum was also transferred to an ultralow temperature freezer for immediate experiments. The protocol for these experiments was approved by the Animal Ethics Committee of Chongqing Medical University and the animal permit number is SYXK (Yu) 2017-0001 (5 March 2017).

### 4.4. Histological Observations

Liver tissues collected from the mice were fixed with 10% buffered formalin, embedded in paraffin, and sectioned to a thickness of 4 μm. Then, the 4-μm sections were stained with hematoxylin and eosin (H&E) following standard protocols. Finally, the histological changes of the stained sections were examined under a light microscopic (Olympus BX43, Olympus Co., Japan), and a representative image in each group was taken.

### 4.5. Measurement of Biochemical Parameters in Serum

Biochemical parameters in serum were determined using commercial diagnostic kits with spectrophotometry (Nanjing Jiancheng Biotechnology Co., Ltd., Nanjing, China), including superoxide dismutase (SOD, A001-2-1), malondialdehyde (MDA, A003-1), glutathione (GHS, A006-1), alanine aminotransferase (ALT, C009-2), and aspartate aminotransferase (AST, C010-2).

### 4.6. RT-qPCR (Real-Time Quantitative Polymerase Chain Reaction) Analyses

Liver tissues collected from the mice were homogenized in a Bioprep-24 homogenizer, and RNA was extracted with Trizol reagent (Invitrogen, Carlsbad, CA, USA). The concentration and purity of RNA was determined using a micro-spectrophotometer (Nano-300, Hangzhou Allsheng Instruments CO., Ltd., Hangzhou, Zhejiang, China). A 1 µg sample of RNA was reverse transcribed to cDNA using a RevertAid First Strand cDNA Synthesis Kit with oligo d(T) primers (Applied Biosystems). The mRNA expression levels of *IL-1β*, *TNF-α*, *Bcl-2*, and *Bax* were measured using a SYBR^®^ Select Master Mix on a Step One Plus Real-Time PCR System (Thermo Fisher Scientific, New York, NY, USA). Fold change was calculated using the 2^−ΔΔCT^ method, and target gene expression levels were normalized to *β-actin*.

### 4.7. Western Blot

Liver tissues collected from the mice were homogenized using a Bioprep-24 homogenizer, and proteins were extracted in RIPA buffer (R0010, Solarbio, Beijing, China) according to the manufacturer’s protocol. Protein concentrations were determined by BCA (Bicinchoninic acid) protein assay kit (PA115, Tiangen, Beijing, China). A 25-μg sample of protein from each group was subjected to SDS-PAGE and transferred to 0.45-µm PVDF membranes (Millipore, Billerica, MA, USA). Membranes were blocked for 2 h at room temperature with 5% skim milk in TBST, and the membranes were respectively incubated with primary antibodies against *Bax* (BS1030), *Bcl-2* (BS1511), *Caspase 3* (BS1518) (Bioworld Technology Inc., Minneapolis, MN, USA), *β-actin* (MA5-15739), and *NF-κB p65* (PA1-186) (Invitrogen, Carlsbad, CA, USA). Secondary antibody was HRP-conjugated secondary antibody (31430, Invitrogen, Carlsbad, CA, USA) in TBST with skim milk. The signaling proteins were detected using chemiluminescence with the enhanced chemiluminescence (ECL) reagent (PE0010, Solarbio, Beijing, China). Band signal intensities were quantified using ImageJ (NIH Image, Bethesda, MD, USA), and normalized to β-actin.

### 4.8. Statistical Analysis

Data are presented as the mean ± standard deviation (SD). Student’s *t*-test was used to determine significant difference between two groups, and more than two groups were compared by ANOVA with Dunnett’s test for post hoc analysis. The analyses were performed with Graph Pad 7.0 (Graph Pad Software Inc., La Jolla, CA, USA).

## 5. Conclusions

Our results demonstrated that L-LF, HK-LF, L-LP, and HK-LP exerted protective effects on improving CCl_4_-induced acute liver injury, but the effects of L-LF or L-LP were significantly better than the corresponding heat-killed group. The effects of HK-LF and HK-LP suggested that their regulating mechanisms are closely related to their cellular components. In addition, the hepatoprotective effects of LF and LP also suggested that they have an important role in liver disease.
